# Supplementary material for: Country-wide medical records infer increased allergy risk of gastric acid inhibition
Source: Nat Commun. 2019 Jul 30;10:3298. doi: 10.1038/s41467-019-10914-6 (PMC6667461; doi:10.1038/s41467-019-10914-6)
Supplement: Supplementary file 1 — Supplementary Material [file 41467_2019_10914_MOESM1_ESM.pdf]

Supplementary Material

Supplementary Table 1. Annual prevalence of anti-allergic and acid inhibitor prescriptions 2009 to 2013 in Austria by gender and age groups

	Anti-allergic medication		Acid inhibitors		Population	
	Males	Females	Males	Females	Males	Females
Age group	%		%		Annual mean	
0-4	7.21	6.01	0.32	0.30	181,101	171,847
5-9	9.09	6.73	0.54	0.59	205,392	195,746
10-14	8.81	6.95	1.76	2.58	216,801	206,949
15-19	7.59	8.32	5.61	8.50	242,036	232,541
20-24	6.23	7.84	8.23	11.21	250,065	247,577
25-29	5.72	7.69	9.97	12.81	270,236	263,871
30-34	5.55	7.81	12.05	14.76	270,625	263,052
35-39	5.71	8.64	14.63	18.03	279,437	278,889
40-44	5.40	8.77	17.02	20.49	329,993	326,802
45-49	4.84	8.31	19.37	23.37	348,506	342,583
50-54	4.43	7.74	22.74	27.48	307,517	308,916
55-59	4.48	7.68	27.50	31.64	249,361	260,934
60-64	4.72	7.92	30.72	35.74	219,734	238,108
65-69	4.84	8.15	34.07	41.10	193,623	220,509
70-74	4.20	7.07	32.25	40.00	180,406	215,711
75-79	4.31	7.38	34.72	44.98	111,622	151,622
80-84	4.14	6.86	31.54	41.93	83,185	136,272
85-89	3.65	5.75	25.64	34.27	41,720	102,670
90+	2.53	3.68	16.32	20.11	14,183	48,105

Supplementary Table 2. ATC/DDD Codes (WHO Anatomical Therapeutic Chemical Classification System with Defined Daily Doses) of analysed substance classes /subclasses

**A ALIMENTARY TRACT AND METABOLISM**	**A02**	**DRUGS FOR ACID RELATED DISORDERS**	***A02A***	***ANTACIDS***	*A02AA*	*Magnesium compounds*
					A02AB	*Aluminium compounds*
					A02AC	*Calcium compounds*
					A02AD	*Combinations and complexes of aluminium, calcium and magnesium compounds*
					A02AF	*Antacids with antiflatulents*
					A02AG	*Antacids with antispasmodics*
					A02AH	*Antacids with sodium bicarbonate*
					A02AX	*Antacids, other combinations*
			A02B	***DRUGS FOR PEPTIC ULCER AND GASTRO-OESOPHAGEAL REFLUX DISEASE (GORD)***	*A02BD*	*Combinations for eradication of Helicobacter pylori*
					A02BA	*H2-receptor antagonists*
					A02BB	*Prostaglandins*
					A02BC	*Proton pump inhibitors*
					A02BX	*Other drugs for GORD (Sucralfate)*
			A02X	***OTHER DRUGS FOR ACID RELATED DISORDERS***		
**R RESPIRATORY SYSTEM**	**R06**	**ANTIHISTAMINES FOR SYSTEMIC USE**	***R06A***	***ANTIHISTAMINES FOR SYSTEMIC USE***	*R06AA*	*Aminoalkyl ethers*
					R06AB	*Substituted alkylamines*
					R06AC	*Substituted ethylene diamines*
					R06AD	*Phenothiazine derivatives*
					R06AE	*Piperazine derivatives*
					R06AK	*Combinations of antihistamines*
					R06AX	*Other antihistamines for systemic use*
**C CARDIOVASCULAR SYSTEM**	**C09**	**AGENTS ACTING ON THE RENIN-ANGIOTENSIN SYSTEM**	***C09A***	***ACE INHIBITORS, PLAIN***	*C09AA*	*ACE inhibitors, plain*
			C09B	***ACE INHIBITORS, COMBINATIONS***	*C09BA*	*ACE inhibitors and diuretics*
					C09BB	*ACE inhibitors and calcium channel blockers*
					C09BX	*ACE inhibitors, other combinations*
			C09C	***ANGIOTENSIN II ANTAGONISTS, PLAIN***	*C09CA*	*Angiotensin II antagonists, plain*
			C09D	***ANGIOTENSIN II ANTAGONISTS, COMBINATIONS***	*C09DA*	*Angiotensin II antagonists and diuretics*
					C09DB	*Angiotensin II antagonists and calcium channel blockers*
					C09DX	*Angiotensin II antagonists, other combinations*
			C09X	***OTHER AGENTS ACTING ON THE RENIN-ANGIOTENSIN SYSTEM***	*C09XA*	*Renin-inhibitors*
	**C10**	**LIPID MODIFYING AGENTS**	***C10A***	***LIPID MODIFYING AGENTS, PLAIN***	*C10AA*	*HMG CoA reductase inhibitors*
					C10AB	*Fibrates*
					C10AC	*Bile acid sequestrants*
					C10AD	*Nicotinic acid and derivatives*
					C10AX	*Other lipid modifying agents*
			C10B	***LIPID MODIFYING AGENTS, COMBINATIONS***	*C10BA*	*HMG CoA reductase inhibitors in combination with other lipid modifying agents combinations*
					C10BX	*HMG CoA reductase inhibitors, other combinations*

Supplementary Table 3. Percentage of individuals with anti-hypertensive or lipid-modifying medications (C09/C10 codes of ATC/DDD) among the total population insured in the county Burgenland and among subgroups with and without acid inhibitor prescription (2009 to 2013) and without non-steroidal anti-inflammatory (M01) co-medication.

Population	N	C09/C10 prescriptions	Percent
All insured	194,765	64,770	33.3
w/o acid inhibitor	89,004	17,890	20.1
with acid inhibitor	105,761	46,880	44.3
Excluding M01 co-medication	51,240	10,609	20.7

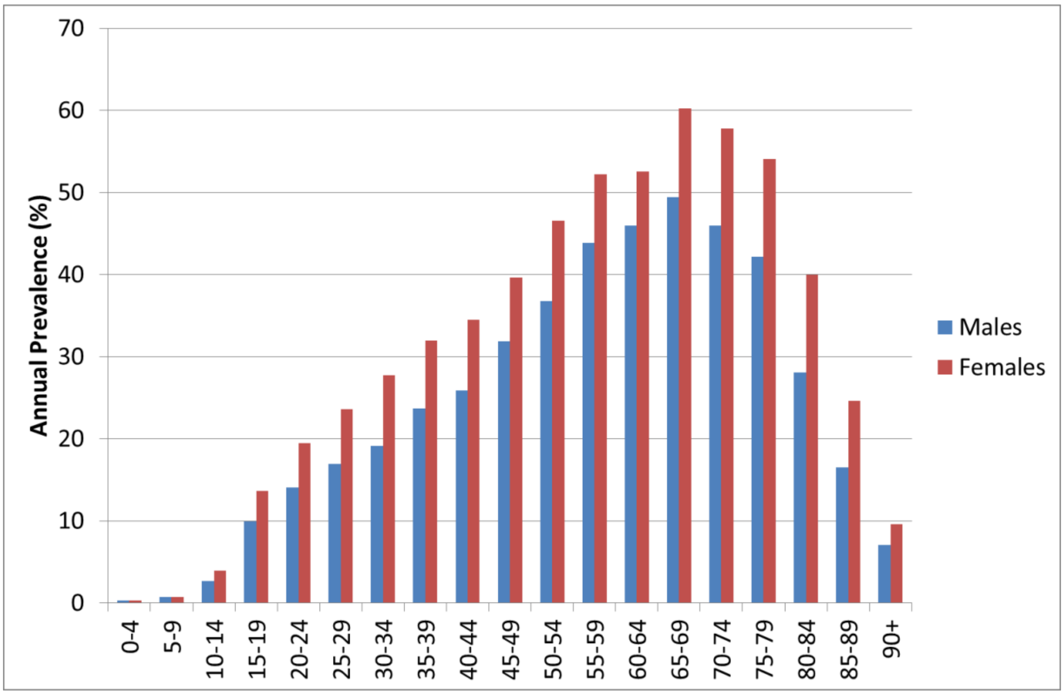

Supplementary Figure 1. Annual prevalence of acid inhibitor prescriptions in the Austrian province Burgenland 2009 to 2013

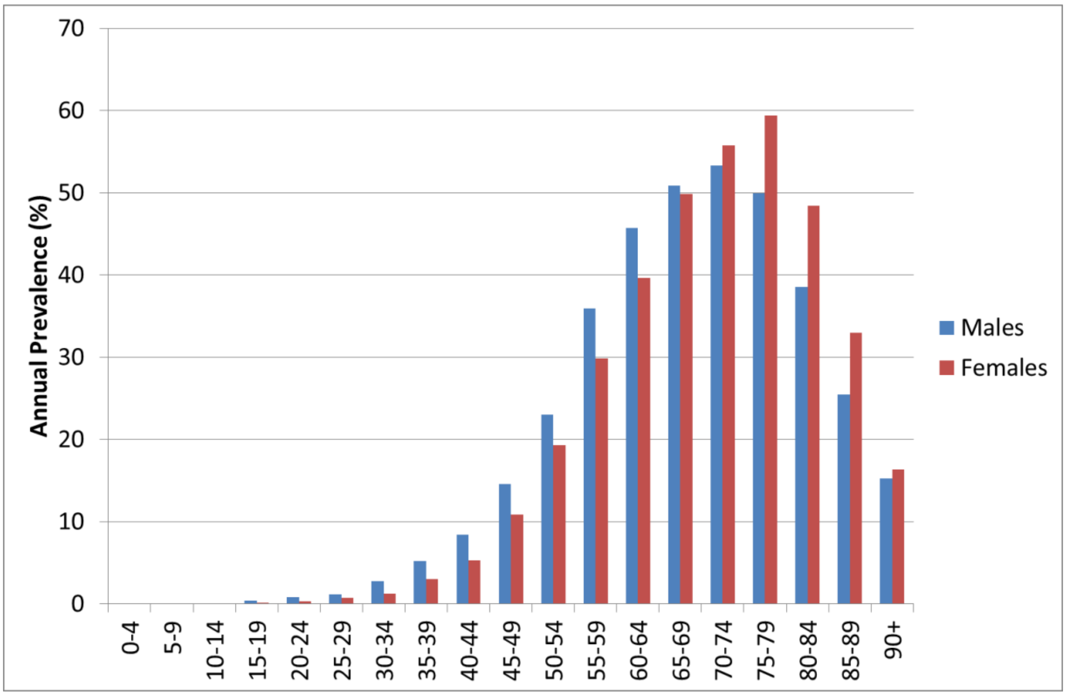

Supplementary Figure 2. Annual prevalence of prescriptions of antihypertensive drugs (C09) in the Austrian province Burgenland 2009 to 2013

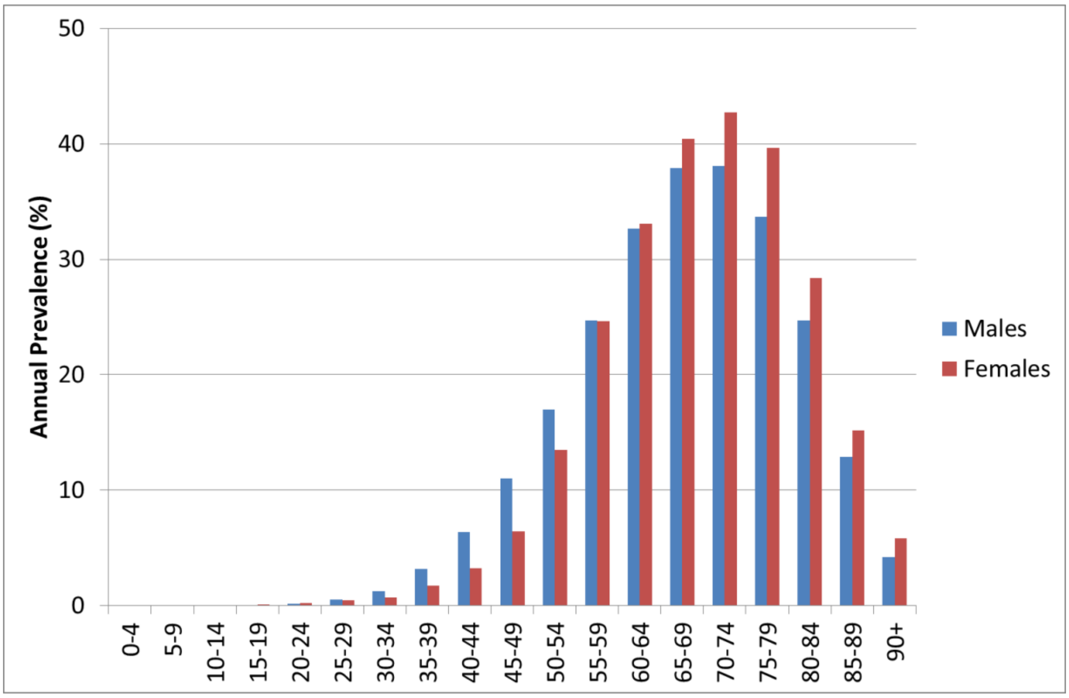

Supplementary Figure 3. Annual prevalence of prescriptions of lipid-modifying drugs (C10) in the Austrian province Burgenland 2009 to 2013

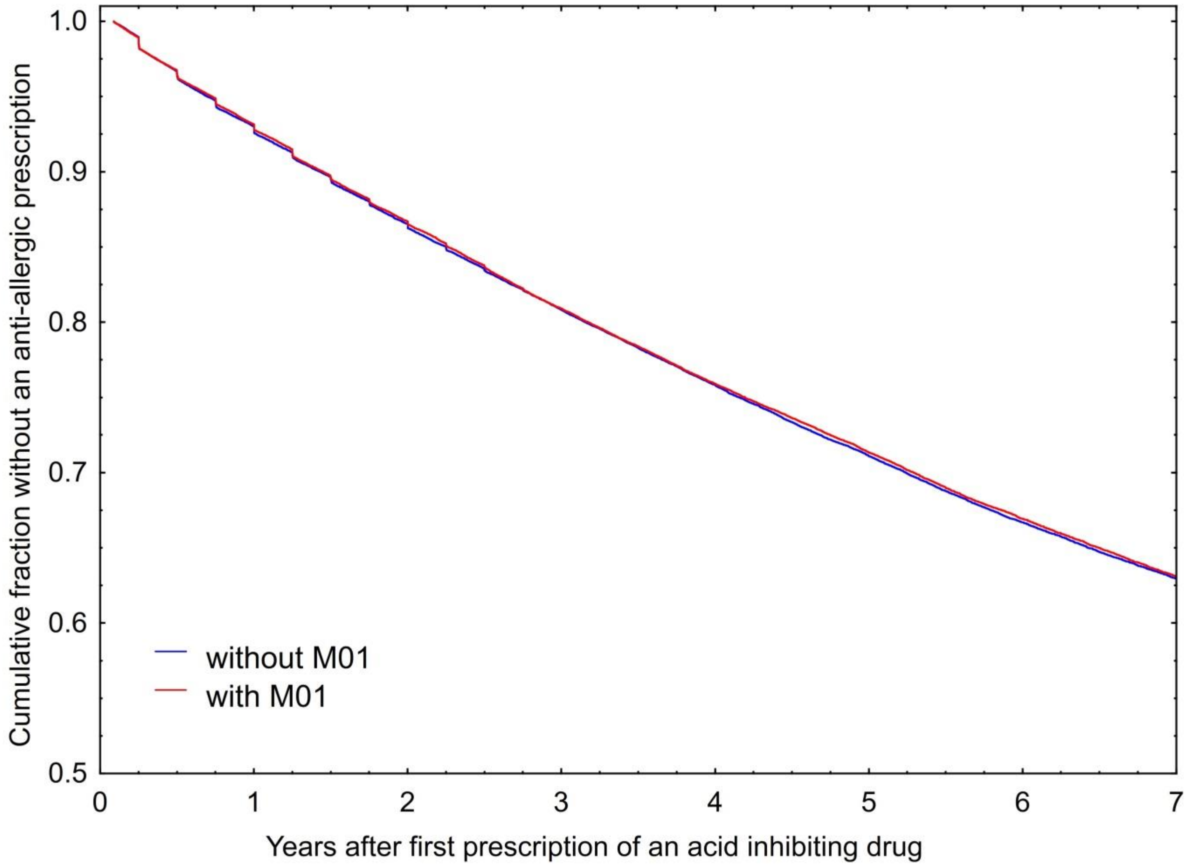

Supplementary Figure 4. Kaplan-Meier plots of the cumulative proportion without subsequent first anti-allergic medication for those with an acid inhibiting drug prescription in individuals with and without non-steroidal anti-inflammatory (M01) co-medication

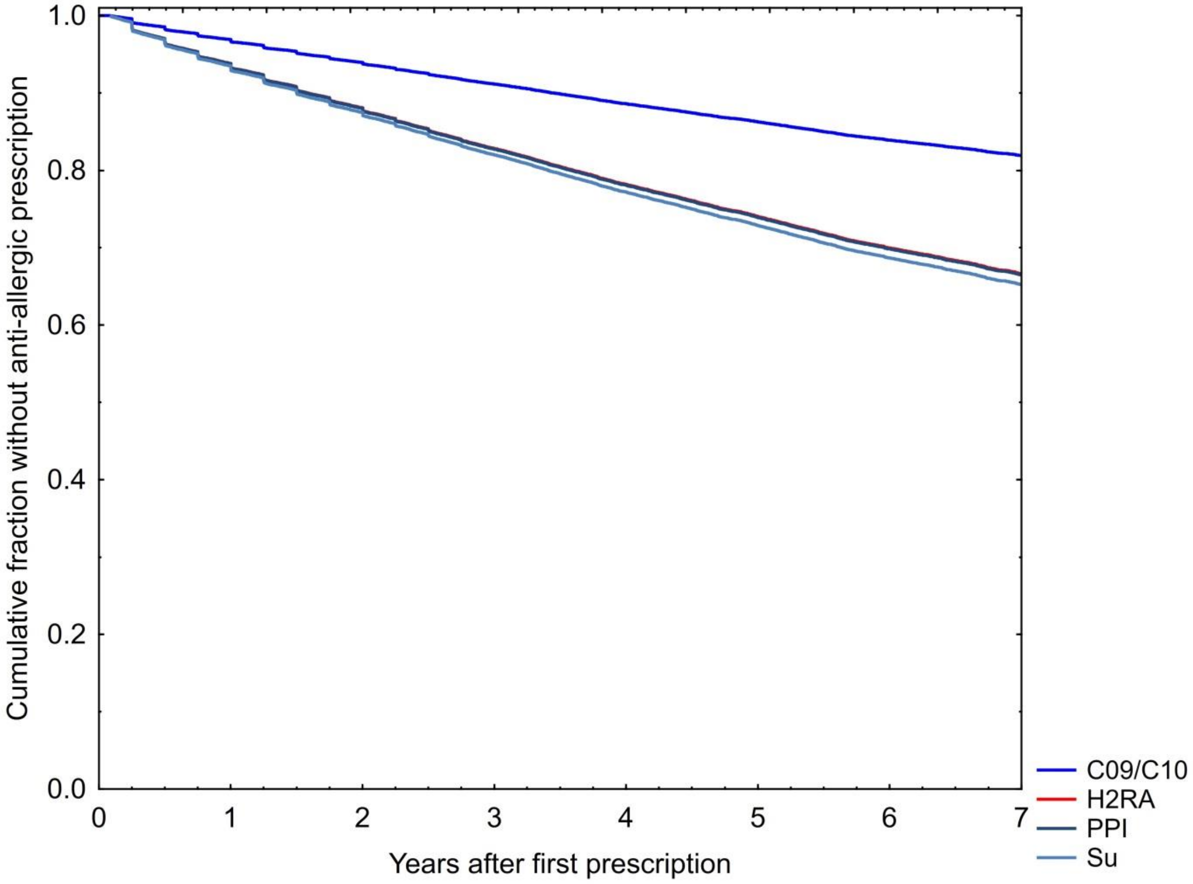

Supplementary Figure 5. Kaplan-Meier plots of the cumulative proportion without subsequent first anti-allergic medication for those with different classes of first acid inhibiting drug prescription and the contrast group of those with an anti-hypertensive or lipid-modifying drug (C09/C10) prescription (H2RA…H2 receptor antagonists, PPI…proton pump inhibitors, Su…sucralfate)

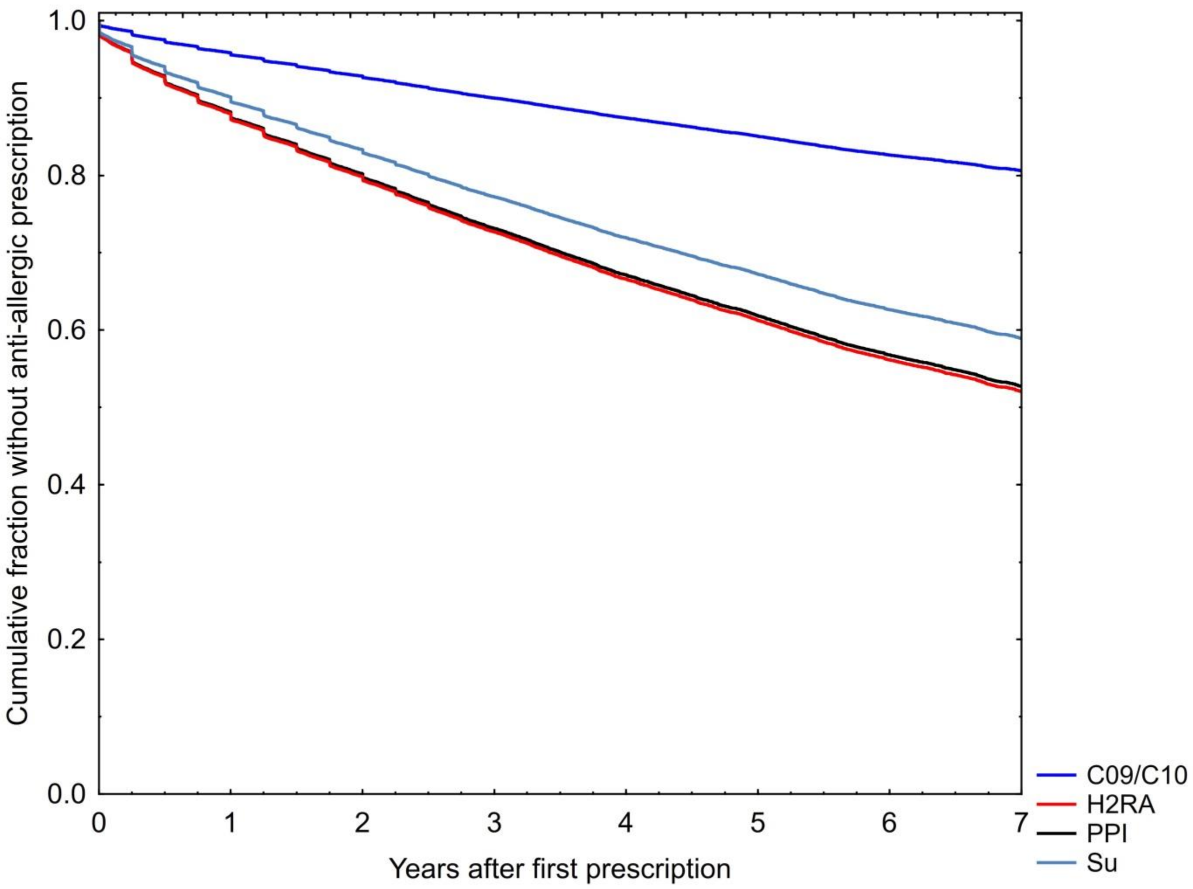

Supplementary Figure 6. Kaplan-Meier plots of the cumulative proportion without subsequent first anti-allergic medication for those with different classes of acid inhibiting drug prescription excluding those with prescriptions from more than one class and the contrast group of those with an anti-hypertensive or lipid-modifying drug (C09/C10) prescription (H2RA…H2 receptor antagonists, PPI…proton pump inhibitors, Su…sucralfate)

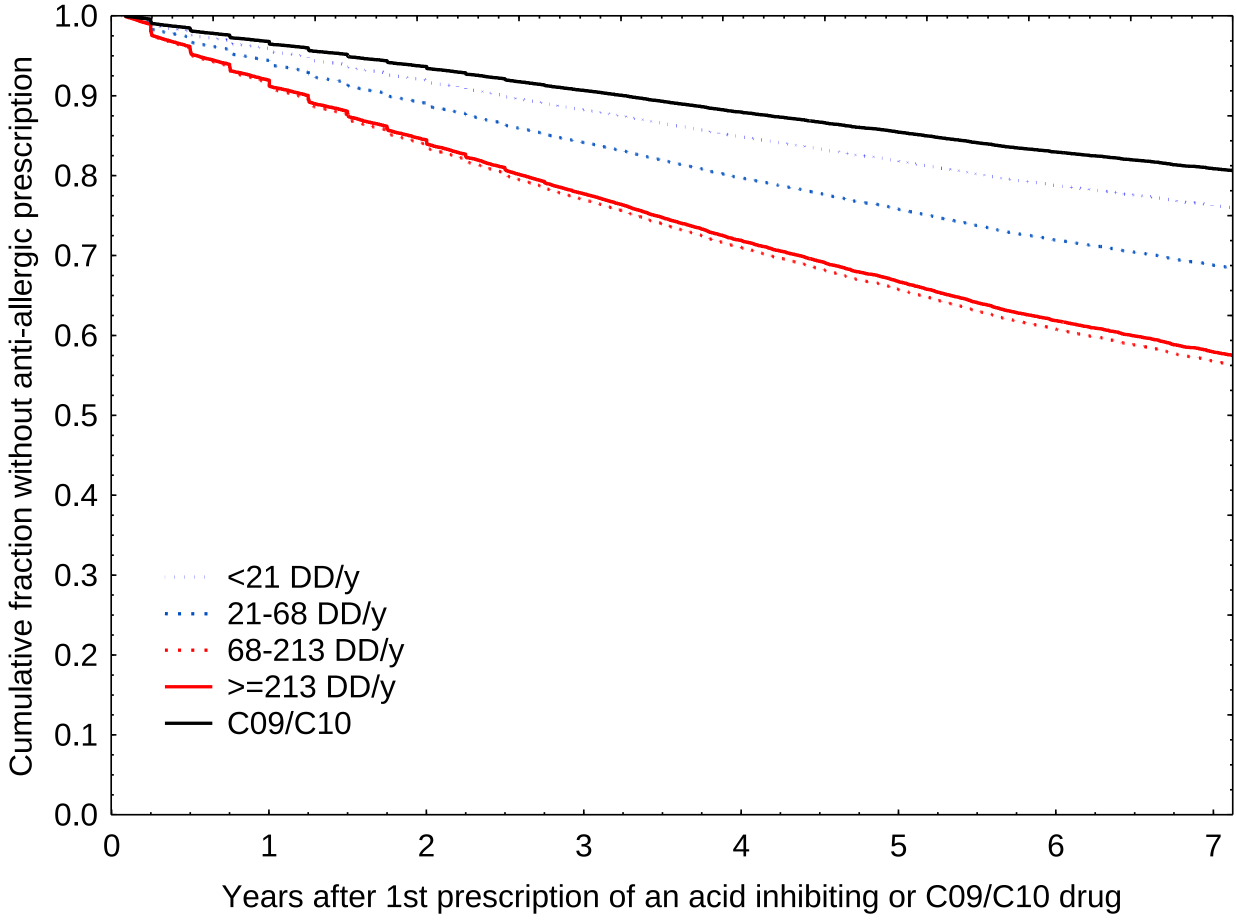

Supplementary Figure 7. Kaplan-Meier plots of the cumulative proportion without subsequent first anti-allergic medication for those with different daily doses per year of an acid inhibiting prescription and the contrast group of those with an anti-hypertensive or lipid-modifying drug (C09/C10) prescription

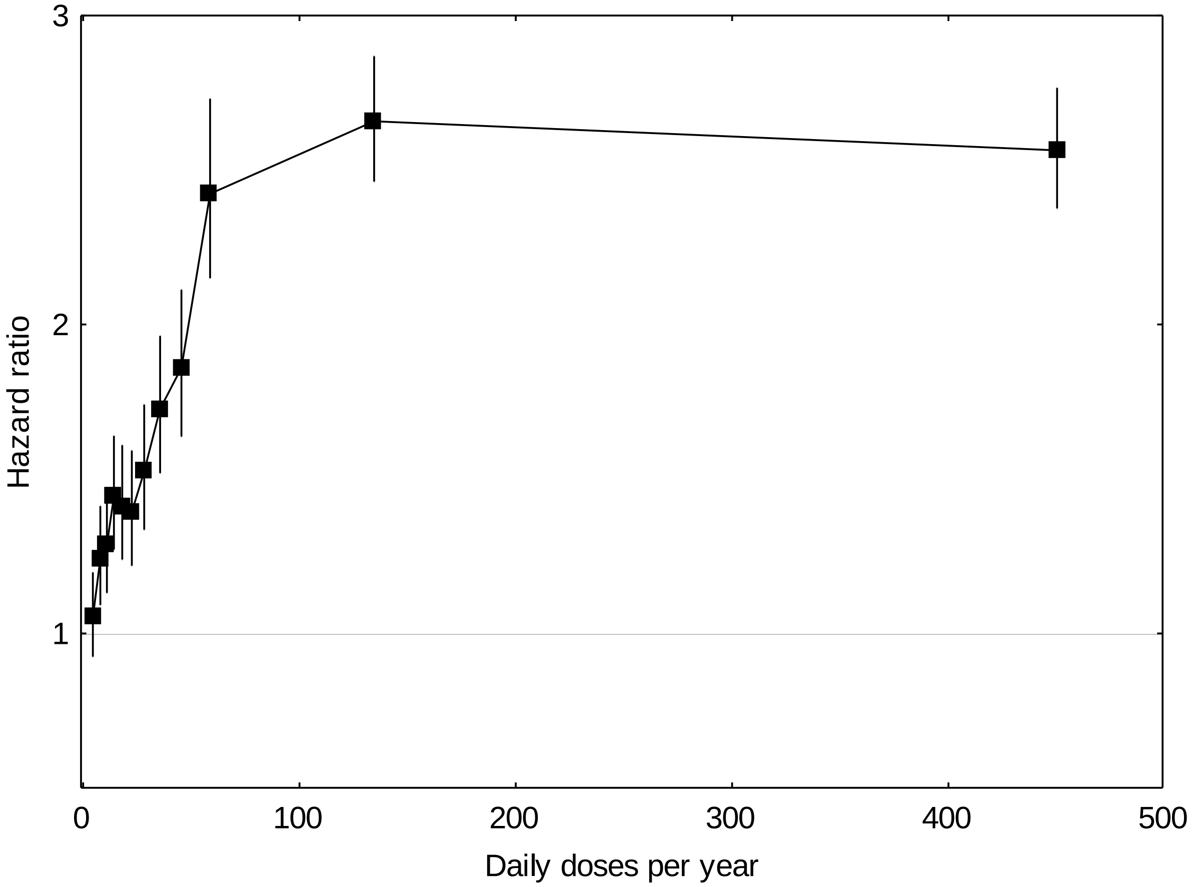

Supplementary Figure S8. Hazard ratios and 95% confidence intervals for categories of daily doses of acid inhibitors with respect to first prescription of an anti-allergic medication
