# Supplementary material for: Country-wide medical records infer increased allergy risk of gastric acid inhibition
Source: Nat Commun. 2019 Jul 30;10:3298. doi: 10.1038/s41467-019-10914-6 (PMC6667461; doi:10.1038/s41467-019-10914-6)
Supplement: Supplementary file 3 — Reporting Summary [file 41467_2019_10914_MOESM3_ESM.pdf]

nature research

Corresponding author(s): Erika JENSEN-JAROLIM

Last updated by author(s): 2019/05/13

Reporting Summary

Nature Research wishes to improve the reproducibility of the work that we publish. This form provides structure for consistency and transparency in reporting. For further information on Nature Research policies, see Authors & Referees and the Editorial Policy Checklist.

Statistics

For all statistical analyses, confirm that the following items are present in the figure legend, table legend, main text, or Methods section.

n/a	Confirmed	
☐	☒	The exact sample size (*n*) for each experimental group/condition, given as a discrete number and unit of measurement
☐	☒	A statement on whether measurements were taken from distinct samples or whether the same sample was measured repeatedly
☐	☒	The statistical test(s) used AND whether they are one- or two-sided<br>*Only common tests should be described solely by name; describe more complex techniques in the Methods section.*
☐	☒	A description of all covariates tested
☐	☒	A description of any assumptions or corrections, such as tests of normality and adjustment for multiple comparisons
☐	☒	A full description of the statistical parameters including central tendency (e.g. means) or other basic estimates (e.g. regression coefficient) AND variation (e.g. standard deviation) or associated estimates of uncertainty (e.g. confidence intervals)
☐	☒	For null hypothesis testing, the test statistic (e.g. *F*, *t*, *r*) with confidence intervals, effect sizes, degrees of freedom and *P* value noted<br>*Give P values as exact values whenever suitable.*
☒	☐	For Bayesian analysis, information on the choice of priors and Markov chain Monte Carlo settings
☐	☒	For hierarchical and complex designs, identification of the appropriate level for tests and full reporting of outcomes
☐	☒	Estimates of effect sizes (e.g. Cohen's *d*, Pearson's *r*), indicating how they were calculated

Our web collection on statistics for biologists contains articles on many of the points above.

Software and code

Policy information about availability of computer code

Data collection	Stata 13.1 (StataCorp, TX, USA) and R 3.1.3 (r-project.org)
Data analysis	Stata 13.1 (StataCorp, TX, USA) and R 3.1.3 (r-project.org)

For manuscripts utilizing custom algorithms or software that are central to the research but not yet described in published literature, software must be made available to editors/reviewers. We strongly encourage code deposition in a community repository (e.g. GitHub). See the Nature Research guidelines for submitting code & software for further information.

Data

Policy information about availability of data

All manuscripts must include a data availability statement. This statement should provide the following information, where applicable:

- Accession codes, unique identifiers, or web links for publicly available datasets
- A list of figures that have associated raw data
- A description of any restrictions on data availability

The data that support the findings of this study were made available to the authors of this study by all major Austrian compulsory health insurance companies under the ethics vote ECS 1134/2014. General Data Protection Regulations Restrictions apply to the availability of these data, which were used under license for this study. Requests for data that support the findings of this study must be therefore addressed in written form to DI Berthold Reichardt, representative for the involved Austrian compulsory health insurance companies.

Field-specific reporting

Please select the one below that is the best fit for your research. If you are not sure, read the appropriate sections before making your selection.

☒ Life sciences ☐ Behavioural & social sciences ☐ Ecological, evolutionary & environmental sciences

For a reference copy of the document with all sections, see nature.com/documents/nr-reporting-summary-flat.pdf

Life sciences study design

All studies must disclose on these points even when the disclosure is negative.

Sample size	Population-based analysis between 2009 and 2013, covering as much of Austria's population as available (8.2 million)
Data exclusions	N/A, No data exclusions
Replication	Experimental findings are based on reproducible calculations from complete data sets provided by all compulsory health insurance companies and were also confirmed in regional subgroup analysis
Randomization	N/A, no experimental groups formed by randomization
Blinding	N/A, investigators conducting data analysis did not participate in data collection, no group allocation during data collection

Reporting for specific materials, systems and methods

We require information from authors about some types of materials, experimental systems and methods used in many studies. Here, indicate whether each material, system or method listed is relevant to your study. If you are not sure if a list item applies to your research, read the appropriate section before selecting a response.

Materials & experimental systems

n/a	Involved in the study	
☒	☐	Antibodies
☒	☐	Eukaryotic cell lines
☒	☐	Palaeontology
☒	☐	Animals and other organisms
☐	☒	Human research participants
☒	☐	Clinical data

Methods

n/a	Involved in the study	
☒	☐	ChIP-seq
☒	☐	Flow cytometry
☒	☐	MRI-based neuroimaging

Human research participants

Policy information about studies involving human research participants

Population characteristics	Population-based analysis (see above), mean age 42.2 years, 48.7 % males, Caucasian
Recruitment	N/A, retrospective insurance claim data analysis
Ethics oversight	The study was approved by the ethics committee of the Medical University Vienna, Austria, prior to commencement (trial registration ECS 1134/2014)

Note that full information on the approval of the study protocol must also be provided in the manuscript.
